# Geology and climate influence rhizobiome composition of the phenotypically diverse tropical tree *Tabebuia heterophylla*

**DOI:** 10.1371/journal.pone.0231083

**Published:** 2020-04-07

**Authors:** Yakshi Ortiz, Carla Restrepo, Brayan Vilanova-Cuevas, Eugenio Santiago-Valentin, Susannah G. Tringe, Filipa Godoy-Vitorino

**Affiliations:** 1 Department of Biology, University of Puerto Rico, San Juan, Puerto Rico; 2 Department of Microbiology and Medical Zoology, University of Puerto Rico, School of Medicine, San Juan, Puerto Rico; 3 Department of Energy, Joint Genome Institute, Walnut Creek, California, United States of America; Universidade de Coimbra, PORTUGAL

## Abstract

Plant-associated microbial communities have diverse phenotypic effects on their hosts that are only beginning to be revealed. We hypothesized that morpho-physiological variations in the tropical tree *Tabebuia heterophylla*, observed on different geological substrates, arise in part due to microbial processes in the rhizosphere. We characterized the microbiota of the rhizosphere and soil communities associated with *T*. *heterophylla* trees in high and low altitude sites (with varying temperature and precipitation) of volcanic, karst and serpentine geologies across Puerto Rico. We sampled 6 areas across the island in three geological materials including volcanic, serpentine and karst soils. Collection was done in 2 elevations (>450m and 0-300m high), that included 3 trees for each site and 4 replicate soil samples per tree of both bulk and rhizosphere. Genomic DNA was extracted from 144 samples, and 16S rRNA V4 sequencing was performed on the Illumina MiSeq platform. Proteobacteria, Actinobacteria, and Verrucomicrobia were the most dominant phyla, and microbiomes clustered by geological substrate and elevation. Volcanic samples were enriched in Verrucomicrobia; karst was dominated by nitrogen-fixing Proteobacteria, and serpentine sites harbored the most diverse communities, with dominant Cyanobacteria. Sites with similar climates but differing geologies showed significant differences on rhizobiota diversity and composition demonstrating the importance of geology in shaping the rhizosphere microbiota, with implications for the plant’s phenotype. Our study sheds light on the combined role of geology and climate in the rhizosphere microbial consortia, likely contributing to the phenotypic plasticity of the trees.

## Introduction

Plant phenotypic plasticity–the capacity to produce more than one phenotype from a single genotype–represents a plant’s response to environmental conditions [1–5], including nutrient availability and climatic conditions such as temperature and moisture [6]. Thus, plants of the same species, growing in contrasting conditions may respond to each situation with phenotypes that increase their fitness [1, 2]. Climate and geology influence the plant’s phenotype both directly and indirectly, including through adaptations achieved by altering the physiology and/or morphology of the plant [7], increasing its survival rates and geographic range [1]. The effect of environmental conditions on plant phenotypic plasticity has been thoroughly studied, although there are other factors modulating the plant phenotype, such as microorganisms associated to plant roots, which just recently gathered scientific attention.

The rhizosphere–a thin layer of soil surrounding plant roots–is influenced by both the root and its associated microbiota (e.g. bacteria and fungi) [8–10], which is recruited by the plant [10, 11]. However, bacteria colonizing the rhizosphere vary due to abiotic factors, including climatic conditions and nutrient availability [9–11]. These abiotic factors contribute to bacterial growth rates, competition (e.g. antibiotic production), and metabolism. The effects of climate and geology on the rhizobiota, and their roles in modulating the phenotypic plasticity of the plant, deserve further attention. With this in mind, we hypothesized that environmental interactions could modify rhizosphere microbial composition and their ecological functions which in turn have an impact in the plant’s phenotype. Our underlying hypotheses are that: 1) climate and 2) geology will independently alter the rhizobiota community composition.

Several groups of microbes alter the plant’s response to the environment such as mycorrhizal fungi, nitrogen-fixing bacteria, and plant growth-promoting bacteria [3, 12–15]. These groups can be beneficial or detrimental to the plant, as they modify rhizosphere processes, which in turn regulate nutrient cycling, water uptake and the response to environmental stress [3, 16, 17]. Therefore, the rhizosphere microbiome has the potential to indirectly increase plant’s fitness by enhancing plant nutrient uptake and altering the plant phenotyp [3]. Our goal was to both characterize the microbial diversity in the rhizosphere of *T*. *heterophylla*, a morphologically variable tree, while identifying influential conditions that may be shaping the rhizosphere microbiota and perhaps their influence on phenotype.

*Tabebuia heterophylla*, or Roble Blanco, is an excellent study subject to test our hypotheses. This plant grows throughout Puerto Rico in a broad altitudinal and geographical range. Exposed to contrasting climates and soils of different parent material, this plant exhibits phenotypic variability in morphological, physiological, reproductive, and growth traits [18]. Notably varying morphological traits such as number and size of leaflets, and size of petioles, are commonly found through the different populations of *T*. *heterophylla* in the island.

To address our hypotheses, we characterized the rhizosphere microbiome of *T*. *heterophylla* under varying geologies and climatic conditions (i.e. temperature and precipitation), to give insight into the extant of the microbial diversity and to further understand the relative role of biotic and abiotic factors in plant phenotypic plasticity.

## Materials and methods

### Sampling design, sample collection, and storage

**Fig 1:**
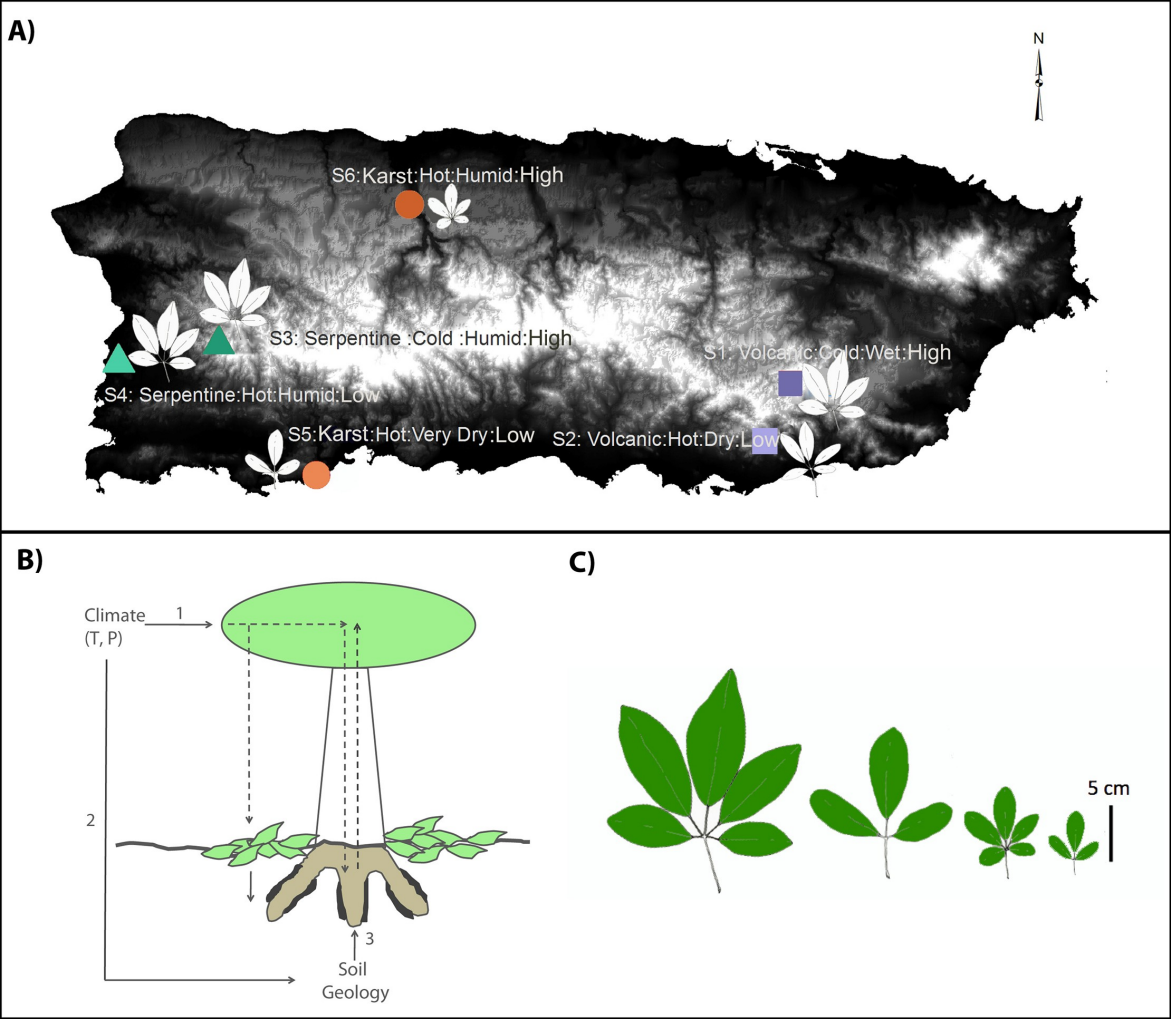
Map of the Island of Puerto Rico with the locations, parental material, temperature, humidity and elevation, and leaf morphology respectively, of each site. GPS coordinates are in Table 1.

*Tabebuia heterophylla* is a tree native to the Caribbean archipelago and is amply distributed in the Island of Puerto Rico. We identified 6 populations of *T*. *heterophylla* developing on soils derived from contrasting parent materials and climatic conditions. For each of the three parent materials (volcanic, serpentine, and limestone/karst formations–henceforth designated as karst) [19], we identified two sites experiencing different climatic conditions (a combination of temperature and precipitation) (Fig 1, Table 1).

**Table 1:** Physical properties of the sites used in this study and their respective number of OTUs and sequences corresponding to sum of all samples (replicates and trees) per site. Analyses henceforth were done with a rarefaction level of 30,170 sequences per sample, corresponding to the sample with the lowest read count.

Site	Location (GPS)	Geology	Elevation (m)	Precipitation (mm)	Temp. (°C)	pH	Read Count		OTU Count	
							Rhizo	Soil	Rhizo	Soil
S1	**Cayey** (18° 06' 28.9'' N 66° 04' 20.7'' W)	Volcanic	647^High^	2077.7^**a**^	21.5^***d***^	5.4	1311511	139907	23478	8480
S2	**Guayama** (18° 01' 24.4'' N 66° 07' 44'' W)	Volcanic	314^Low^	1661.4^**b**^	23.9^**c**^	6	1041329	694638	21825	21013
S3	**Maricao** (18° 09' 33.4'' N 66° 59' 55.5'' W)	Serpentine	711^High^	2392.9^**a**^	23.1^**c**^	7.1	781076	402700	31430	21925
S4	**Cabo Rojo** (18° 07' 51.1" N 67° 09' 34.4" W)	Serpentine	70^Low^	1352.0^**c**^	25.3^**b**^	6.5	219426	565129	16444	22466
S5	**Guanica** (17°58′18″N 66°54′29″W)	Karst	10^Low^	783.7^**d**^	26.3^**a**^	8.7	2323394	1172332	30242	25240
S6	**Arecibo** (18° 22' 49.1'' N 66° 41' 09.6'' W)	Karst	250^High^	1864.9^**b**^	24.5^**b**^	7.3	3326940	1832653	39446	32406

^a^ Very high

^b^ High

^c^ Medium

^d^ Low

Site 1 (S1 Fig) was located in a high altitude volcanic area in Cayey (Bosque de Carite), 18° 06' 28.9'' N 66° 04' 20.7'' W; Site 2 (S2) was located in a low altitude area in Guayama 18° 01' 24.4'' N 66° 07' 44'' W. Serpentine sites corresponded to sites 3 and 4, Site 3 (S3) was in the high altitude Bosque Estatal de Maricao 18° 09' 33.4'' N 66° 59' 55.5'' W; and Site 4 (S4) was in Cabo Rojo, low altitude (near sea level), 18° 07' 51.1" N 67° 09' 34.4" W. Limestone/karst areas (in this work, karst) were located in Bosque Estatal de Guánica (low altitude karst) 17°58′18″N 66°54′29″W and in high altitude area, Arecibo (S6), 18° 22' 49.1'' N 66° 41' 09.6'' W. Thus, each site represents a unique combination of parent material and climate (Table 1). A map representing the collection sites, was prepared using the SRTM–Shuttle Radar Topography Mission Digital Elevation Data (90 m). At each site, three *T*. *heterophylla* trees (each tree represents a sampling unit) were chosen based on similar sizes, to sample the rhizosphere and nearby bulk soil. Once the tree was identified, the soil was dug nearby to identify the main root and from there, further digging was done—in a depth from 1–1.5 foot to 5 feet deep- until we could collect secondary roots branching out from the main root. For each tree, four secondary roots (each root per tree represents a subsample) extending between 46–91 cm were collected, cut and stored in a 50ml falcon tube prior to being transported in ice to the laboratory. Additionally bulk soil close to every tree was also collected. At each of four points ~ 30.5 cm from each selected tree, soils were sampled down to 15 cm with a soil auger. The bottom 10 cm of the soil samples were stored in sterile 50 ml Falcon tubes for transport from the field to the laboratory. Sample collection followed protocols from a previous study [20], and was performed under permit number DRNA 2011-IC-018, issued by the Puerto Rico Department of Natural Resources, to the Biology Department of the University of Puerto Rico-Río Piedras Campus.

In the lab, soil samples were immediately frozen at -80°C until gDNA extraction, while roots were washed with 25ml PBS/Tween20 and shaken at 240 rpm horizontally for 1 hour. After roots were bare, we removed them from the PBS solution and froze them in new Falcon tubes at -80°C. The PBS/Tween20 solution with the rhizosphere was centrifuged at 9,000g for 30 min at 4°C. The resulting pellet with the rhizosphere was frozen at -80 C until DNA extraction.

### Genomic DNA extractions and 16S rRNA amplifications

Rhizosphere and bulk soil samples were processed for metagenomic DNA extraction and amplification of 16S rRNA genes. The genomic DNA was extracted from the rhizosphere and bulk soil subsamples (4 subsamples per individual for a total of 24 subsamples per site, or 144 subsamples in total), using the PowerSoil DNA isolation kit (MO BIO, Carlsbad, CA, USA) following the manufacturer’s instructions. The genomic DNA samples were sent to the Joint Genome Institute of the Department of Energy for amplification of the V4 hypervariable region (~291bp length) of the 16S ribosomal RNA (rRNA) and sequencing was performed using the Illumina MiSeq platform following an in-house protocol.

Itag data can be downloaded from the JGI repository (https://genome.jgi.doe.gov/portal/pages/dynamicOrganismDownload.jsf?organism=Tabitaplate2_FD).

### Community profiling analyses

The raw reads were subjected to pre-processing, using JGI’s pipeline iTagger, which performed read QC (filter contaminants such as PhiX control, sequencing library adapter dimers, human contaminants using Duk). Sequence clustering was done with USEARCH [21] including sequence cleaning and identity assignment. Clustering was done iteratively starting at 99% identity, and decreasing by 1% identity until the level described in the config file is reached (97%). Finally, we used Quantitative Insights Into Microbial Ecology (QIIME) pipeline versions 1.8 and 1.9 [22] for downstream sequence processing such as chimera removal taxonomic classification to each cluster using RDP Classifier. From the 144 subsamples, only 137 samples were analyzed considering only samples with more than 1,000 reads. Lastly, sequences corresponding to chloroplast DNA (plant DNA) were removed from the OTU table through a taxa filtering function. For some of the analyses, we collapsed 137 subsamples through the collapse function of QIIME v1.9 [22], by summing the sequences from all subsamples per tree, which is our sampling unit (n = 36). We selected the minimum number of sequences of collapsed samples, (30, 170 sequences; to rarefy all samples and subsequently perform the statistical analyses.

### Statistical analyses

For each site we extracted the total annual precipitation (bio12) and mean annual temperature (bio01) from BIOCLIM data set using ArcGIS 10.7 [23]. The two climatic variables were converted into categorical variables each with four levels (very high, high, medium, and low; Table 1), which together with the parent material (volcanic, serpentine and karst) were used to build a mapping file. Our sequence data and this mapping file were inputted to QIIME v1.9 pipeline [22] and the Phyloseq R package [24].

We examined variation in species’ richness (OTUs) across the parent material and climate matrix based on the Chao 1 index estimated by QIIME [22] and visualized through R [25]. The error bars represent confidence intervals (95%, df = 9, two tail T values) that were calculated using 10 iterations of the Chao1 index of the subsamples, depending on the case. We also examined variation in species diversity through Shannon index and visualized through Phyloseq [24]. Analyses of Variance tests, implemented in R (50),were used to find significant differences in alpha diversity related to a given category. Additionally, differences among categories, i.e combinations of microhabitat/ sample type (rhizosphere and bulk soil), geology and climate, were tested with an ANOVA and Tukey HSD posthoc test for all variables and were considered significant at an FDR-corrected p < 0.01. We ran two complementary analyses to examine variation in community structure (beta diversity). The first used the subsamples to run a non-metric multidimensional scaling (nMDS) ordinations based on a Bray Curtis distance matrix using ggplot2 [26]. The second, used the collapsed samples into sampling units in a Principal Coordinate Analysis (PCoA) ordination based on a Unifrac distance matrix and visualized through Phyloseq [24].

Measures of beta diversity were assessed for differences between sample groups using two different complementary non-parametric analyses for multivariate data analysis of similarity (ANOSIM) [27] and nonparametric multivariate analysis of variance (Adonis) using distance matrices [28]. We used the Bray–Curtis similarity index to calculate a distance matrix for ANOSIM and Adonis, and performed the analyses with the Vegan package [29] in the R package using R [25].

We also generated taxa summaries with QIIME [22], filtering only OTUs present in at least 40% of the samples using the script compute_core_microbiome.py, and visualized using R [25]. Biomarker discriminant taxa analyses were performed with the LEfSe algorithm [30] for multi-class comparison. In order to reduce false discovery rates, FDR p-values <0.01 were considered for significant differences—these analyses was performed using the MicrobiomeAnalyst pipeline [31].

## Results and discussion

We collected rhizosphere and bulk soil samples from 18 *Tabebuia* trees that grew in soils of different geologies (i.e. volcanic, serpentine and karst). Per each geology, we chose two sites that differed in elevation, and we selected three *T*. *heterophylla* trees per site to collect our samples (Table 1).

Trees were sampled in four geographically and climatically varying sites (Fig 1A).

Sites 1 and 2 had more acidic pH and corresponded to volcanic sites of high and low altitude, respectively (Table 1). Sites 3 and 4 had a neutral-like pH and corresponded to serpentine sites of high and low altitude and Sites 5 and 6 corresponded to more alkaline pH of karst origin of low and high altitude respectively (Table 1).

The 18 trees used to characterize *T*. *heterophylla’s* rhizosphere microbiome yielded ~13.8 million non-chimeric 16S rRNA sequence reads with an average of 383,640 sequences per sample. These reads represented a total of 36,601 OTUs belonging to 69 different phyla. Rarefication of the samples to a uniform depth of 30,170 sequences reduced the total OTUs observed to 26,075, with an average of 3,591 OTUs observed per sample and reduced the number of phyla to 62. Table 1 summarizes the number of sequences and OTUs per site before and after the rarefaction. Analyses considering only OTUs present in at least 40% of the samples resulted in 26 Phyla.

**Fig 2:**
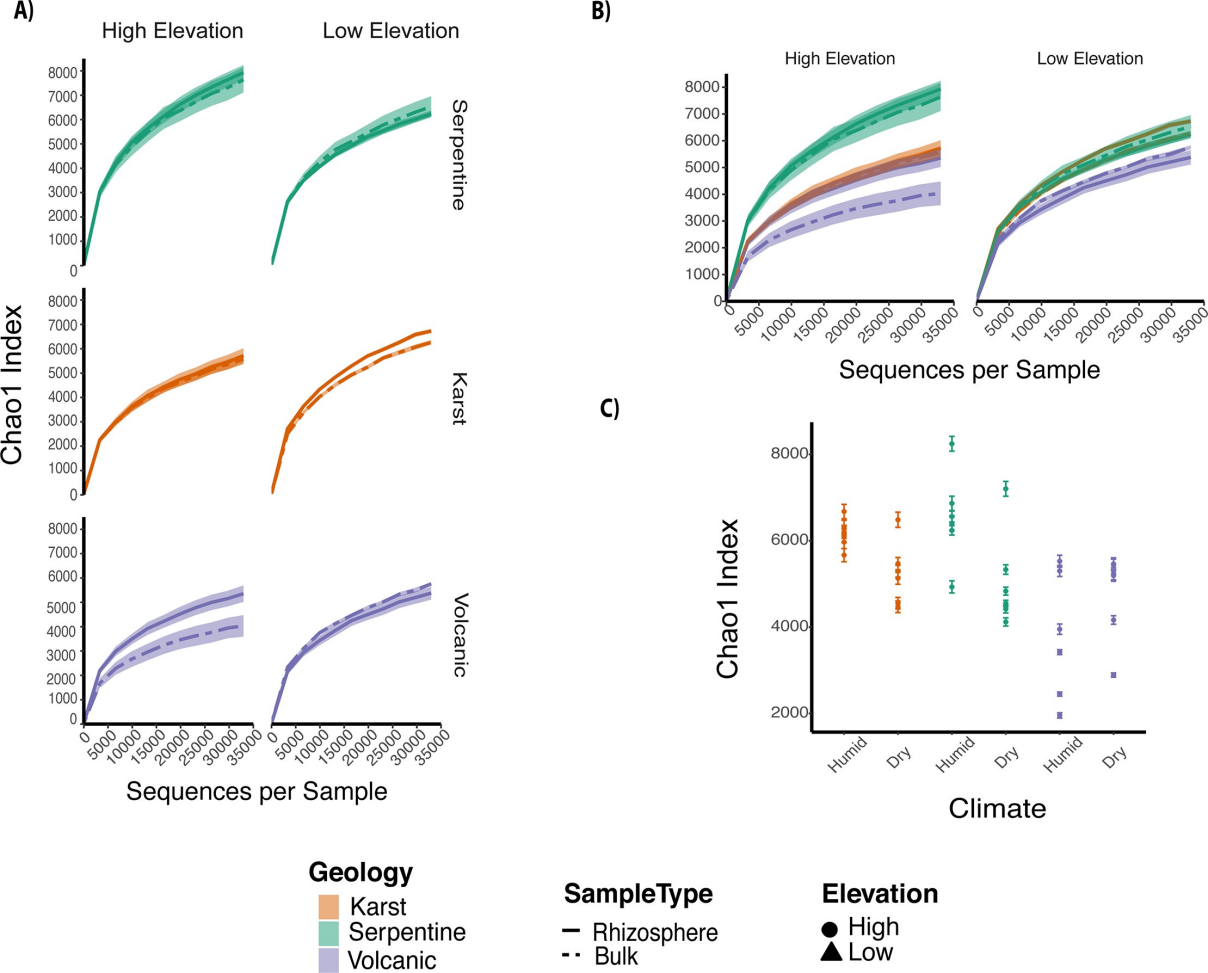
Alpha diversity plots depicting richness estimations. Panel A shows rarefaction curves of rhizosphere and soil microbial communities for each of three parent materials. Panels are organized from sites with highest to the lowest number of OTUs (serpentine to volcanic) and columns show sites with high and low elevation. Curves were constructed using Chao1 index for samples with up to 30,170 reads. Bars reflect the 95% confidence intervals. Panel B depict rarefaction curves organized by elevation, whereas in panel C samples are color coded by geology while x-axis corresponds to humid and dry climate for each of the three geologies.

Species richness (Chao 1) of rhizosphere and bulk soil was greatest in serpentine (with neutral pH)- in both high and low elevation, followed by karst and volcanic sites (Fig 2A and 2B). Serpentine sites were significantly richer compared to volcanic (p-value <0.05), but not significantly higher than Karst, except for high altitude, humid sites, where richness was significantly higher in serpentine sites, compared to karst (S1 Table). The same differences were found regarding Shannon diversity with decreased diversity in volcanic sites (p-value <0.05) (S1 Fig). Overall, rhizosphere samples possessed significantly higher species richness than bulk soil samples (p-value <0.05), though per sample site this was not the case (S2 Fig, S1 Table). The individual trees at each site didn’t have a defined variability pattern (S2 Fig); sites where rhizosphere had low variability could have either low or high bulk soil species richness, although in most cases the rhizosphere was more diverse than the bulk soil at each of the individual trees (S2 Fig).

Within geology, we found less species richness in low elevation sites, where temperature was higher and precipitation was lower (Table 1), especially for bulk soil compared to rhizosphere (Fig 2A, S2 Fig). Indeed, trees in the humid serpentine site had the highest richness of all samples (Fig 2C) and significantly different from all other geological sites at both altitudes. Additionally, volcanic bulk soil samples had significantly different diversity between low and high elevation sites (p-value = 0.00), while there were no differences in the rhizosphere communities of these same sites (p-value = 1.000) (S2 Fig, S1 Table).

**Fig 3:**
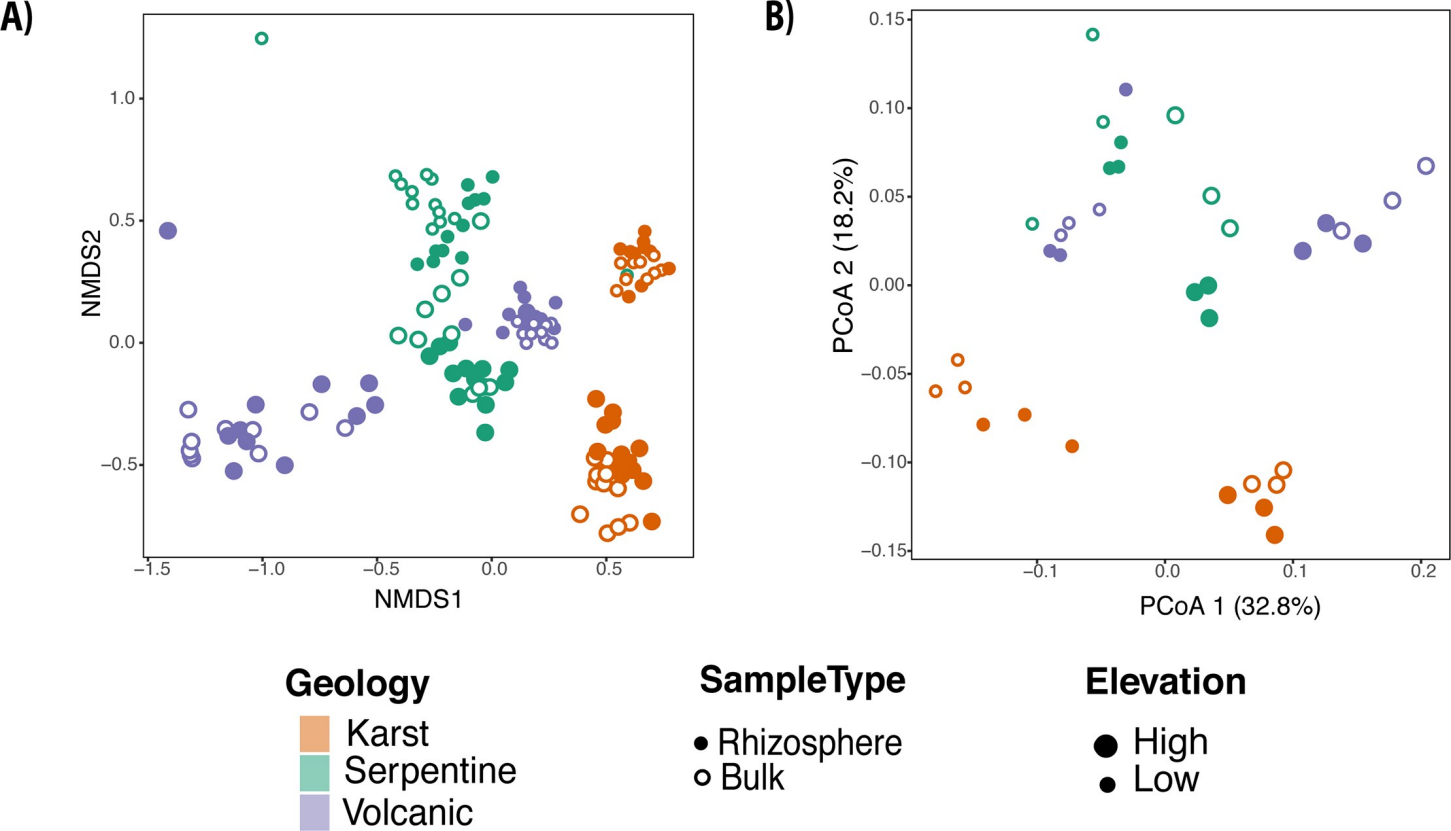
Beta diversity plots. Panel A shows nMDS ordination based on the relative dissimilarities of the 137 samples (Bray Curtis) (stress 0.1232491) representing each parent material and elevation along with microhabitat. The Principal coordinates of panel B, corresponding to the collapsed sample units n = 36, demonstrate that primary clustering is by geology and elevation, mainly separating the Karst samples from the rest of the parent materials as shown also in the nMDS plot.

In general, the rhizosphere and bulk soil bacterial communities within each site clustered separately both in nMDS and in Principal Coordinates Analysis (Fig 3A and 3B respectively). Primary clustering was by samples of sites under same geology (ANOSIM statistic R: 0.569, p-value = 0.001). In fact, karst samples (regardless of altitude and microhabitat) clustered together and separated from the rest of the parent materials (NMDS1). Clustering also occurred in samples from similar environmental conditions (elevation) (ANOSIM statistic R: 0.3888, p-value = 0.001) (Fig 3A). Rhizosphere and bulk soil samples of the low elevation karstic site (S5) were clearly separated from other sites, while the high elevation volcanic site (S1 Fig), was in the opposite extreme of the nMDS ordination. This is potentially caused by the temperature and moisture gradient from the hot and dry low elevation karst site, to the colder and wetter high elevation volcanic site (Fig 3A; Table 1). Combined replicates per tree(n = 36), along sites, also display a separation along PCoA Axis 1(Fig 3B); karstic sites (S5 and S6) were located at the negative side of the axis, while serpentine sites (S3 and S4) were at the most positive side of Axis 2 (Fig 3B). This arrangement along Axis 2 suggests a possible effect of the parent material, although serpentine and volcanic bacterial communities did not separate completely.

We do not discard a potential influence of the annual mean temperature as well. Soil and rhizosphere samples from low altitude karst, which showed a separation from all other samples, have a unique leaf phenotype, composed of three leaflets, abovate and rounded, while all other trees had 5 leaflets (Fig 1, S3A Fig, S3B Fig). The leaves of the low elevation karst are the only ones with a rounded apex with no drip tip, while all others were acute with a drip tip for rapid water shedding (S3C Fig). Karst samples, from both humid and dry sites, cluster together phenotypically since they have the shortest leaflet petiole length (S3D Fig), and have the smallest trees (S3E Fig). As explained before, samples from dry karst (site 5) correspond to very high temperatures and cluster separately from other temperatures with the low temperature site being the most dissimilar (S3F Fig). Even though the temperature range differences may seem small, combined with differences in other factors, like precipitation or water availability, can result in considerably different microenvironments that can affect the biology of some plant species.

**Fig 4:**
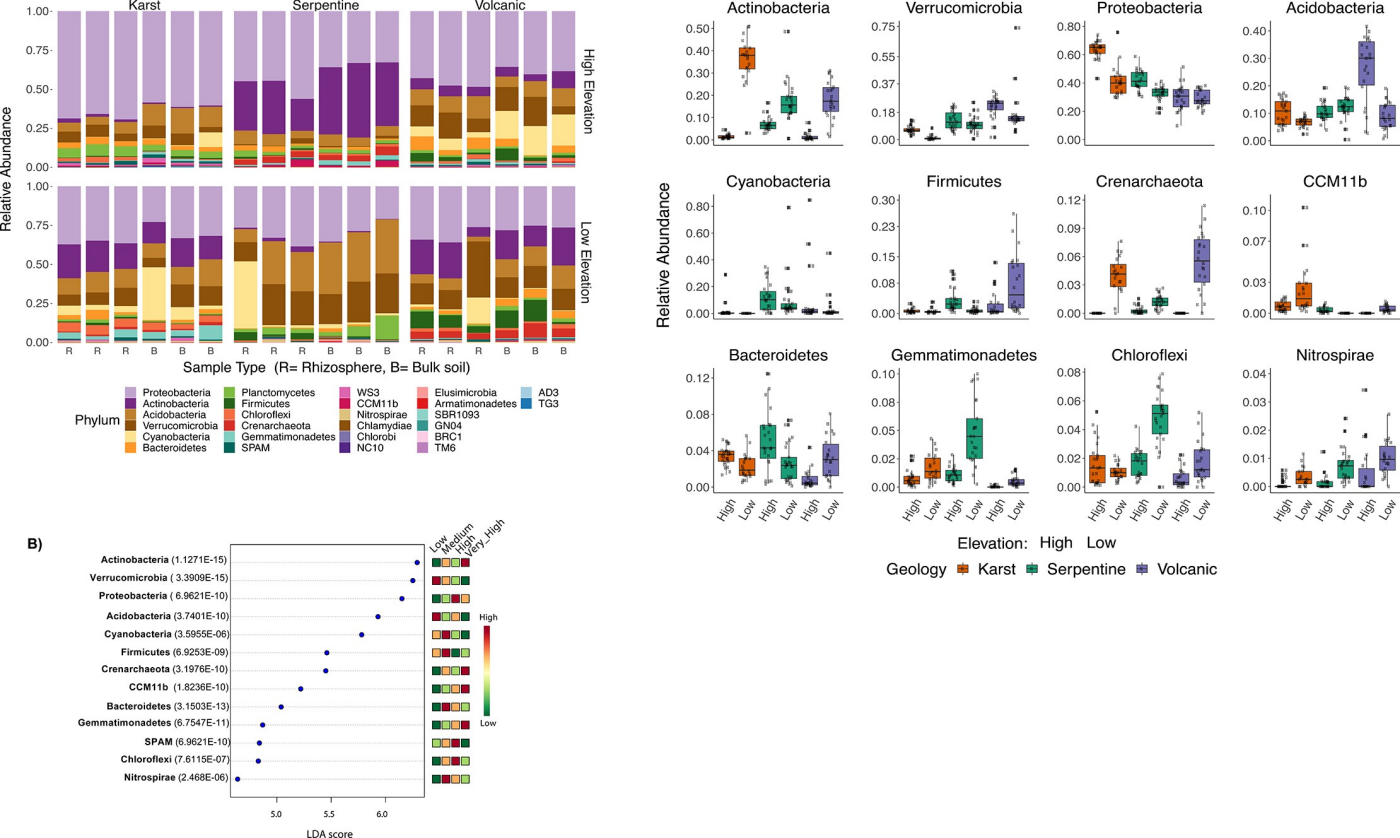
Composition of the bacterial communities across soil and rhizosphere samples per geology and altitude. Panel shows a phyla-level barplot, with samples sorted by geology and elevation considering only OTUs present in at least 40% of the samples, and with relative abundances greater than 3%. Panels B and C show significant different phyla across geology; panel B shows discriminant phyla-level analyses (FDR p-values <0.05) according to geology and altitude, calculated with LEfSe (Linear discriminant analysis Effect Size) algorithm. The algorithm employs non-parametric Kruskal-Wallis rank sum test to detect phyla with significant differential abundance for geology, and panel B shows discriminating taxa per geology and altitude.

Patterns of taxon abundance helped further elucidate the microbial communities’ differences across sites. Focusing on prevalent OTUs present in at least 40% of the samples and with relative abundances greater than 3%, 26 Phyla were observed among which the most dominant were Proteobacteria, followed by Actinobacteria and Acidobacteria; the only archaeal phylum accounting for >10% of the community was Crenarchaeota (Fig 4A). A total of 13 phyla changed significantly with the mean annual temperature (Fig 4B, S4 Fig). Indeed, sites with lower temperatures had significantly higher amounts of Verrucomicrobia and Acidobacteria, especially in the high elevation volcanic site (S1 Fig) (Fig 4C). The high elevation serpentine site (S3) which had medium temperatures, site resulted with significantly higher amounts of Cyanobacteria, Firmicutes, Nitrospirae, and Bacteroidetes than all other sites (Fig 4B and 4C). High temperature sites (including both karst sites) had significantly higher amounts of Proteobacteria (Fig 4B), SPAM (now called Candidate phylum Rokubacteria [32]) and Chloroflexi, while sites with very high temperature had significantly higher amounts of Actinobacteria (as did dry sites of all geologies to some extent), Crenarchaeota (mostly in dry karst and volcanic sites) and CCM11b (mostly in the dry karst) (Fig 4B, S4 Fig). Elevation (and humidity) also affected phylum-level abundances, with low elevation sites exhibiting a greater abundance of Actinobacteria, Acidobacteria, and Gemmatimonadetes (S4C Fig).

**Fig 5:**
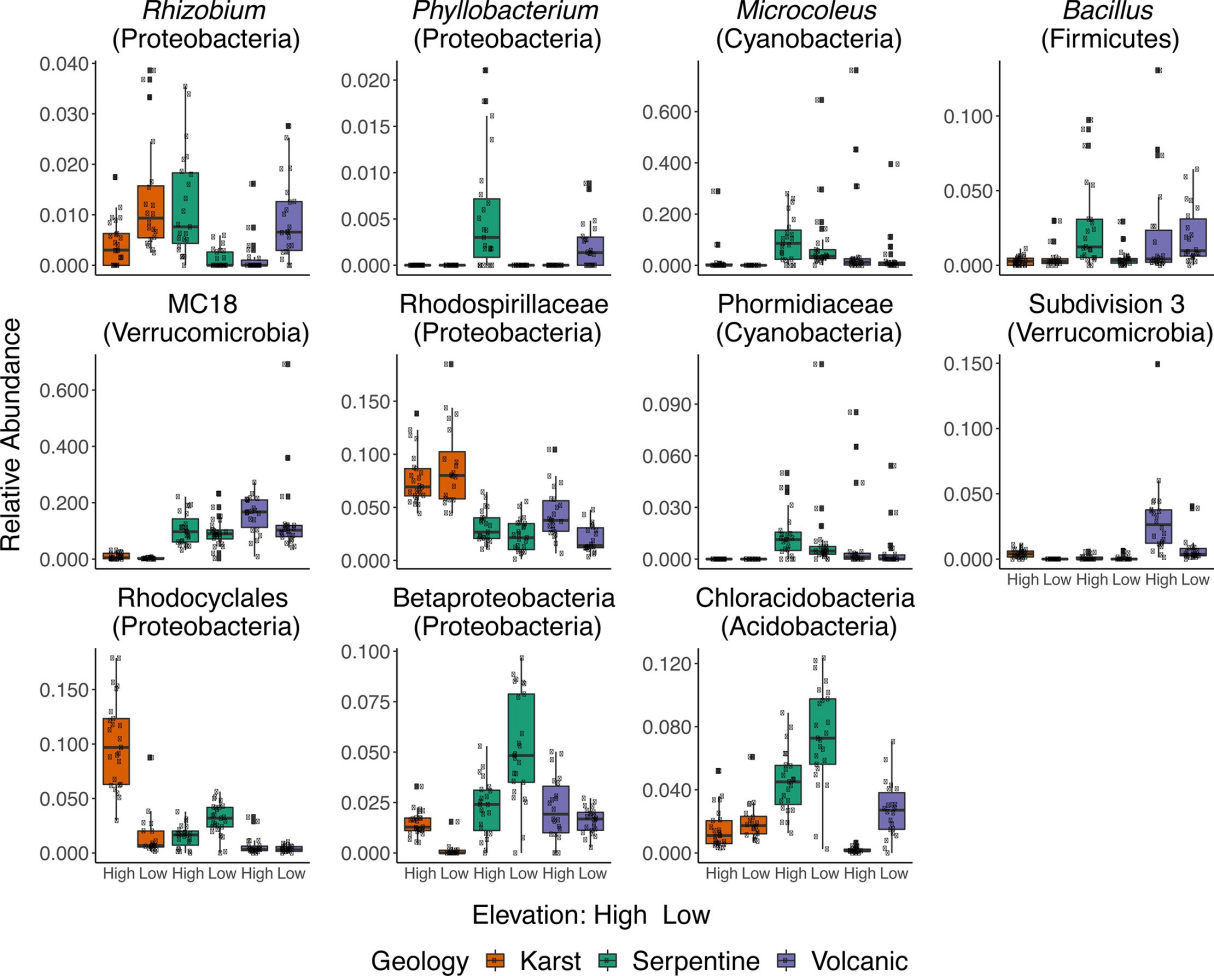
Boxplots of significantly discriminant taxa-level analyses according to both geology and altitude, via LEfSe (Linear discriminant analysis effect size) algorithm. Taxa shown here correspond mostly to genus-level or to the lowest unclassified taxa. Phyla are in parenthesis.

Effects of geology and elevation were more apparent at lower taxonomic levels (class and genus). At the parent material scale, volcanic sites are enriched in Verrucomicrobial genus MC18 and Rhodoplanes (Fig 5). Karst sites harbor significantly more Rhodocyclales, CCM11b, Sinobacteraceae while at serpentine sites Cyanobacteria (*Microcoleus)*, Acidobacteria (Chloracidobacteria) and *Bradyrhizobium* (Fig 5, S5 Fig).

Dry karst sites had significantly higher read abundance of OTUs within order Rhodocyclales, *Rubrobacter* or *Solirubrobacter* while humid karst sites had significantly higher amounts of *Rhizobium*, CCM11bPH and OTUs within family *Rhodospirillaceae*. Serpentine high elevation sites had significantly higher amounts of OTUs in class Betaproteobacteria, *Chloracidobacteria*, and *Phyllobacterium*. Drier serpentine sites had significantly higher quantities of certain Cyanobacteria, such as *Microcoleus* and OTUs within family Phormidiaceae (Fig 5,S5 Fig). Volcanic dry sites had higher dominance of MC18 (Verrucomicrobia)—both greater abundance among all genus, as well as higher abundances in volcanic sites compared other geologies. Other genus dominant in Volcanic dry sites, *Bacillus* and Verrucomicrobia_subdivision_3 (Fig 5, S5 Fig).

While there were no consistent differences between rhizosphere and bulk soil across all sites, there were some differences regarding the composition of bacteria between rhizosphere and bulk soil samples within every parent material type. These differences included a greater abundance of *Microcoleus* in bulk soils compared to rhizosphere at serpentine sites; increased *Bradyrhizobium* in the rhizosphere of serpentine and volcanic sites relative to matched bulk soils; or elevated *Rhizobium* in the rhizosphere of karst samples compared to bulk soils (S6 Fig).

The analysis of *Tabebuia* rhizosphere and bulk soil communities exhibited patterns of taxa that were related to climate (temperature, elevation), geology (parent material), microhabitat (i.e. rhizosphere or bulk soil), and phenotypic features such as leaf morphology.

Comparison of the rhizosphere and bulk soil microbial communities showed significantly higher species richness in the former than in the latter, although these differences were not significant at site level. In addition, there were slightly different species composition per sample type/microhabitat. Our PCoA ordination revealed clusters by sites, with sub-clusters by microhabitat suggesting that rhizosphere microbial communities are distinct from those of the bulk soil at all geologies. These results are not surprising since the micro-conditions of the rhizosphere are distinct from those of the soil. For instance, rhizosphere holds higher nutrients and water levels than the soil [33], which probably allows different bacterial and archaeal species to colonize. Indeed, we found taxa characterized by being nodulating nitrogen-fixing bacteria in significant abundance in rhizosphere compared to soils. Differences in composition and abundance between rhizosphere and bulk soil communities was also found in arctic soils [34]. Most bulk soils are more diverse than rhizosphere but this is likely true in nutrient-replete soils, compared to tropical soils where frequent rainfall may leach nutrients away; indeed, drier sites had higher bulk soil diversity while sites of high elevation had more diverse rhizospheres.

We hypothesized that environmental interactions could modify rhizosphere microbes and their functions, which in turn would have an impact in the plant’s phenotype. Indeed, we found that the most distinct microbiomes, as shown in the beta diversity plots, were from low altitude karst which had very high mean annual temperature and low precipitation; these correspond to the shortest trees, with markedly different leaf morphology. Seminal evidence from other studies suggests that microbiomes alter plant phenotypic plasticity in a broad range of traits [35] and common garden experiments also suggest a role of plant genotypes shaping its microbiota and the microbes in turn participate in the development of plant phenotypes [36].

To test the hypothesis that climate will alter the rhizosphere microbial communities, we focused on two sites of the same geology but with the most contrasting climatic conditions. Serpentine sites (S3 and S4 Figs), which had a difference in the mean annual temperature of 2.2°C and 1041 mm of rain, had significantly higher rhizosphere diversity between both sites and compared to other sites. However, their microbial community compositions aren’t notably different from each other, except for the greater abundance of Chloracidobacteria and Betaproteobacteria in the high elevation site. These differences in the microbial communities can be ascribed to differences in the environmental conditions at the different altitudes, emphasizing the importance of variables such precipitation and temperature, as previously shown in other geographical studies [37, 38]. Indeed, studies have observed greater environmental fluctuations in low altitude sites, whereas high altitude sites have normally lower temperature, nutrient, and water availability [39–41]. Indeed a recent study found selective proliferation of Gram negative bacteria at high altitude, including psychrophilic diazotrophs, along with a decrease in Gram positive bacteria [41], however these studies were done at significantly higher altitudes than those assessed in this manuscript.

For the hypothesis that geology will alter the microbial community in the rhizosphere, having an indirect effect over the phenotype, we chose sites with similar climatic conditions but originating from different parental material. Sites 2 (volcanic) and 6 (karst) possess a difference of only 0.6°C and 203.5mm of rain (Table 1), and differ significantly in their diversity levels, as well as in their microbial community composition. These results suggest that geology may have a greater effect on the microbial community composition of the rhizosphere as well as on the leaf phenology/phenotype, than other environmental factors.

Changes in microbial community composition across sites were related to temperature, precipitation, pH, and geology. The highest diversity was observed in the serpentine samples, which possess ~neutral pH and temperatures near 24°C. This diversity peak is consistent with a previous study of geothermal environments, where the highest diversity was also observed in samples collected at pH 7 with temperatures of 24°C [42]. As in their study, we also found that diversity declined as the temperatures became more extreme. However, our data found pH was a strong diversity-driving factor, which is consistent with other studies [43–45], and mean annual precipitation also appears to be a key driver of microbial communities found across the different sites.

The rhizosphere is a thin layer of soil surrounding the plant root, influenced by the root and its associated microbiota [8–10]. Plants produce an array of different exudates (e.g. amino acids, organic acids, polysaccharides) used by bacteria and archaea to produce energy [8, 10]. Thus, the rhizosphere will be dominated by microbes with the metabolic capacity to degrade those compounds in addition to other structural compounds such as lignin, commonly found within the plant litter [46, 47]. These degradation processes are essential for plant nutrient cycling, [48] biogeochemical cycles (e.g. nitrogen, phosphorus, calcium) and water uptake [49]. Several groups of microbes are known to alter the plant response to the environment [3], such as mycorrhizal fungi, nitrogen-fixing bacteria, and plant growth-promoting bacteria (PGPB). These groups alter the rhizosphere processes, which in turn regulate different aspects of nutrient cycling and water uptake, promoting the establishment of plants in stressed environments [3, 16]. Our study found a higher diversity of Verrucomicrobial OTUs in Volcanic sites, taxa well known to be glycosyl hydrolase rich heterotrophs, adapted to the utilization of sulfated glycopolymers such as those from subdivision 3 and soil clone MC18 [50]. Serpentine sites are known to be nutrient-poor and high in heavy metals such as chromium, nickel, and cobalt [51]. We found locally adapted serpentine microorganisms in the rhizosphere of plants corresponding to dominance of Cyanobacteria such as *Microcoleus* and Phormidiaceae OTUs. Cyanobacteria have been shown to be able to tolerate the toxicity of heavy metals. Heavy metals are well known to cause generation of reactive oxygen species (ROS) and reactive nitrogen species (NO) which are involved in the free radical chain reaction of membrane lipids and proteins [52]. Metal homeostasis is a special property of Cyanobacteria, as the photosynthetic machinery demands metals that act as cofactors of several proteins and other types of photosynthetic metabolism [53]. Additionally, the adaptation to metal concentrations in Cyanobacteria includes their ability to excrete heavy metal siderophores [54], the production of metalloproteins and metal transporters [55] and tolerance of a significant concentration of heavy metals by reducing the toxic effect of ROS with several antioxidant enzymes [53, 56]. Along with these, Chloracidobacterial OTUs were also found in high abundance in serpentine sites, which are also photosynthetic [57]. In the karst bedrock sites -known to be porous with underground drainage systems due to the rainfall–loss of nitrogen is common [58]. We found a higher abundance of *Rhizobium*, Rhodocyclales, Rhodospirillaceae *and* CCM11b OTUs in both karst sites in high abundance among all other genera and also in higher abundance compared to the other volcanic and serpentine sites. These taxa are anoxygenic photoheterotrophs; plant-associated nitrogen-fixing aerobes, and non-sulfur photosynthetic bacteria [59].

Despite growing in stressful heavy-metal rich serpentine soils, *Tabebuia* trees select for serpentine tolerant bacteria and show a similar phenotype as nutrient-rich volcanic-adapted trees. Karst-adapted trees, especially those in very dry and hot climates, have the most distinct phenotype, although with a similar microbial diversity index and different microbial composition; thus this extreme phenotype may well be a result of adaptation to aridity [60]. Our results suggest that climate and geology impact the rhizosphere microbial pool with consequences for their function and to the plant phenotype. Tabebuia microbiomes thus can indirectly improve the plant’s fitness (e.g. nutrient availability), which may ultimately result in an altered phenotype.

## Supporting information

S1 Table Statistical alpha diversity comparisons between rhizosphere and soil communities in each geologic and climatic site. (XLSX)

S1 Fig Dotplot of alpha diversity of combined bulk soil and rhizosphere samples, according to geology and precipitation. (PDF)

S2 Fig Rarefaction curves of bacterial diversity per tree according to geology and elevation, comparing rhizosphere and bulk soil samples. (PDF)

S3 Fig PCoA plots depicting phenotypes. Panel A shows microbiomes according to leaflet number; panel B compared leaflet shape, panel C shows apex morphology, panel D shows petiole length, panel E shows a range of tree height and Panel F displays microbial communities based on mean annual temperature.
(PDF)

S4 Fig Discriminant phyla-level analyses according to mean annual temperature (x-axis), with LEfSe (Linear discriminant analysis effect size) algorithm. The algorithm employs non- parametric Kruskal-Wallis rank sum test to detect phyla with significant differential abundance for a given category (mean annual temperature).
(PDF)

S5 Fig Discriminant genus changing in abundance according to geology, using LEfSe. (PDF)

S6 Fig Discriminant genus-level analyses according to geology and microhabitat/sample type. (PDF)
