# Supplementary figures and images for: Geology and climate influence rhizobiome composition of the phenotypically diverse tropical tree Tabebuia heterophylla
Source: PLoS One. 2020 Apr 7;15(4):e0231083. doi: 10.1371/journal.pone.0231083 (PMC7138329; doi:10.1371/journal.pone.0231083)

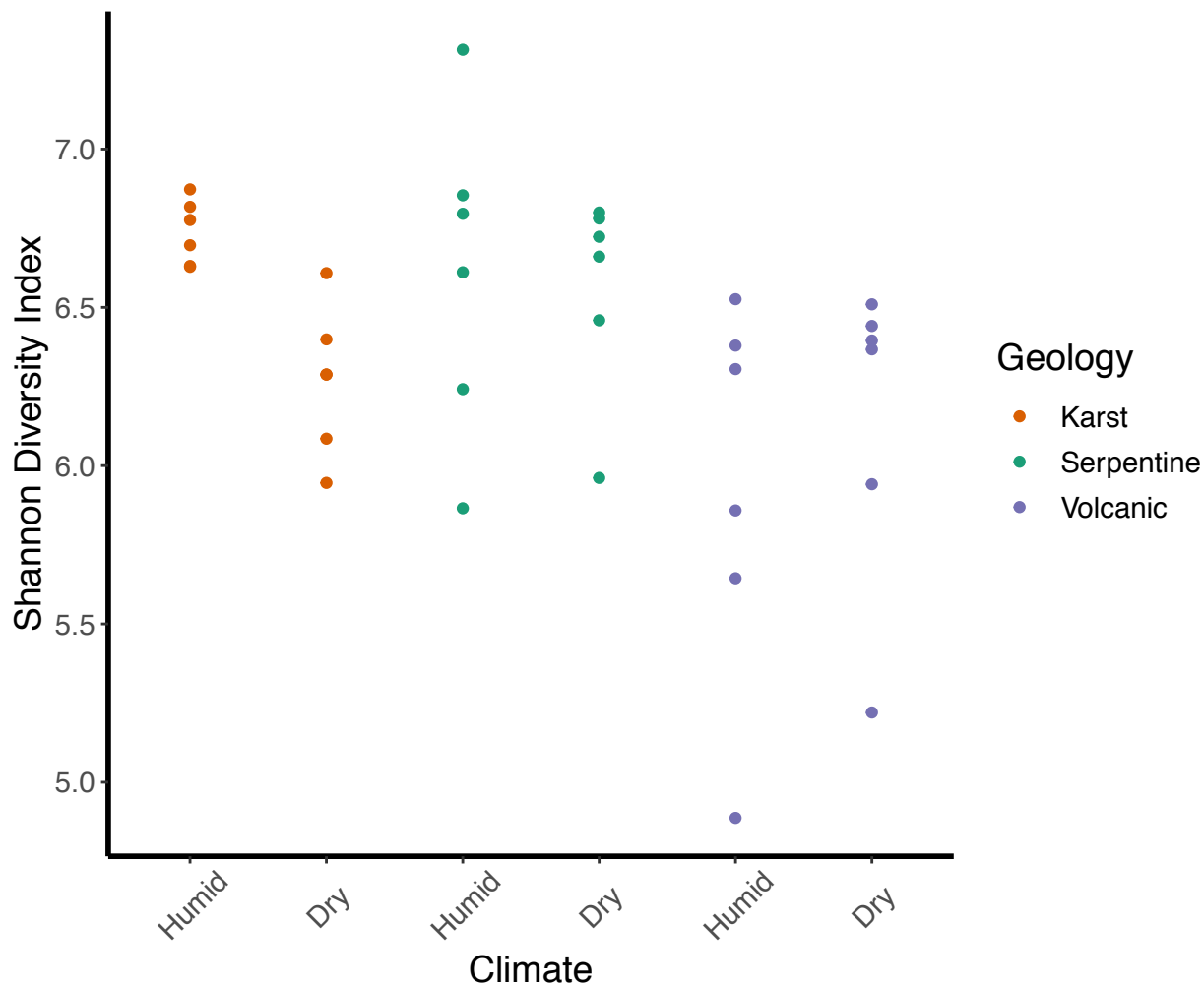

p-value=2.9494e-16,  
F-value22.53

Supplement: S1 Fig — (PDF) [file pone.0231083.s002.pdf]

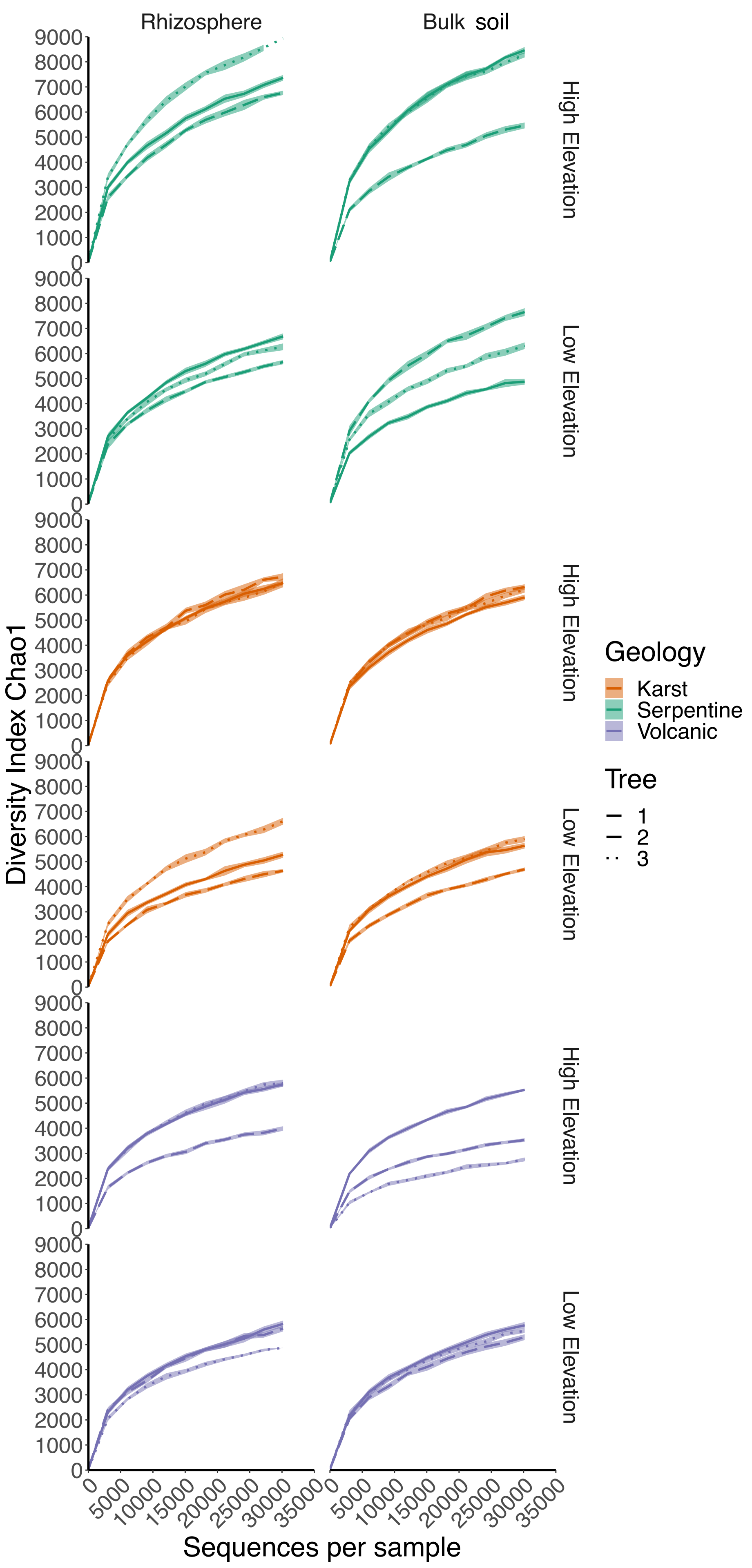

Supplement: S2 Fig — (PDF) [file pone.0231083.s003.pdf]

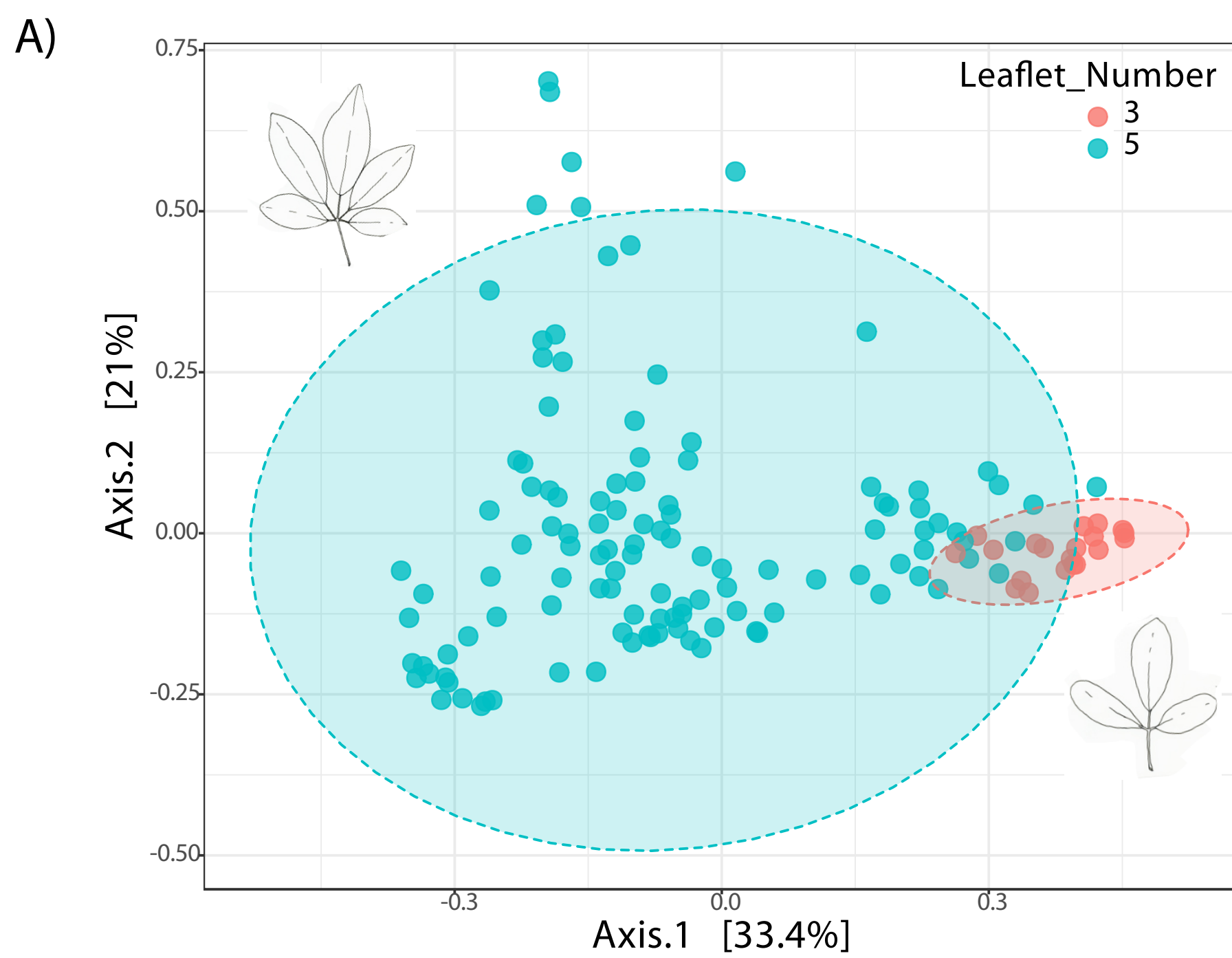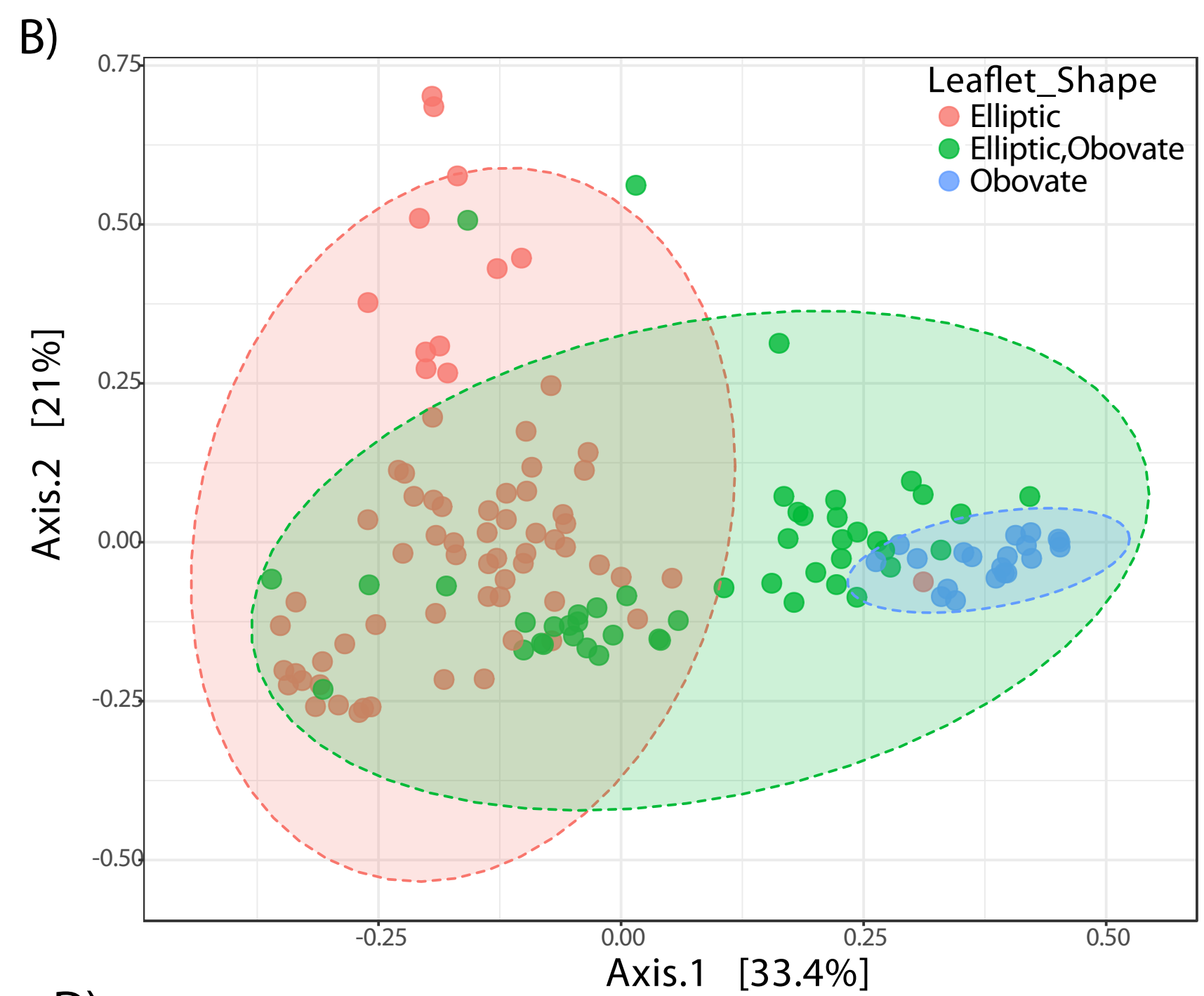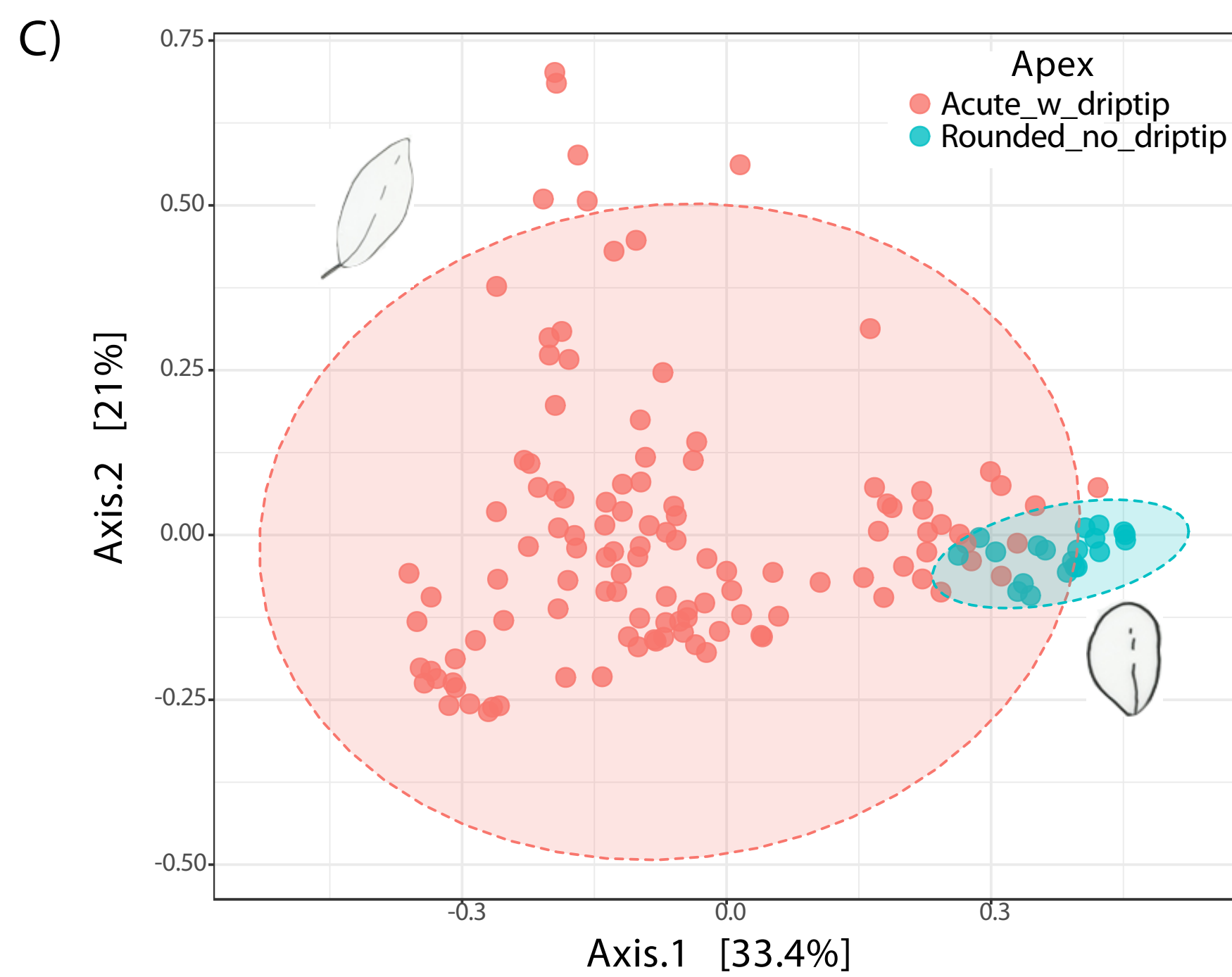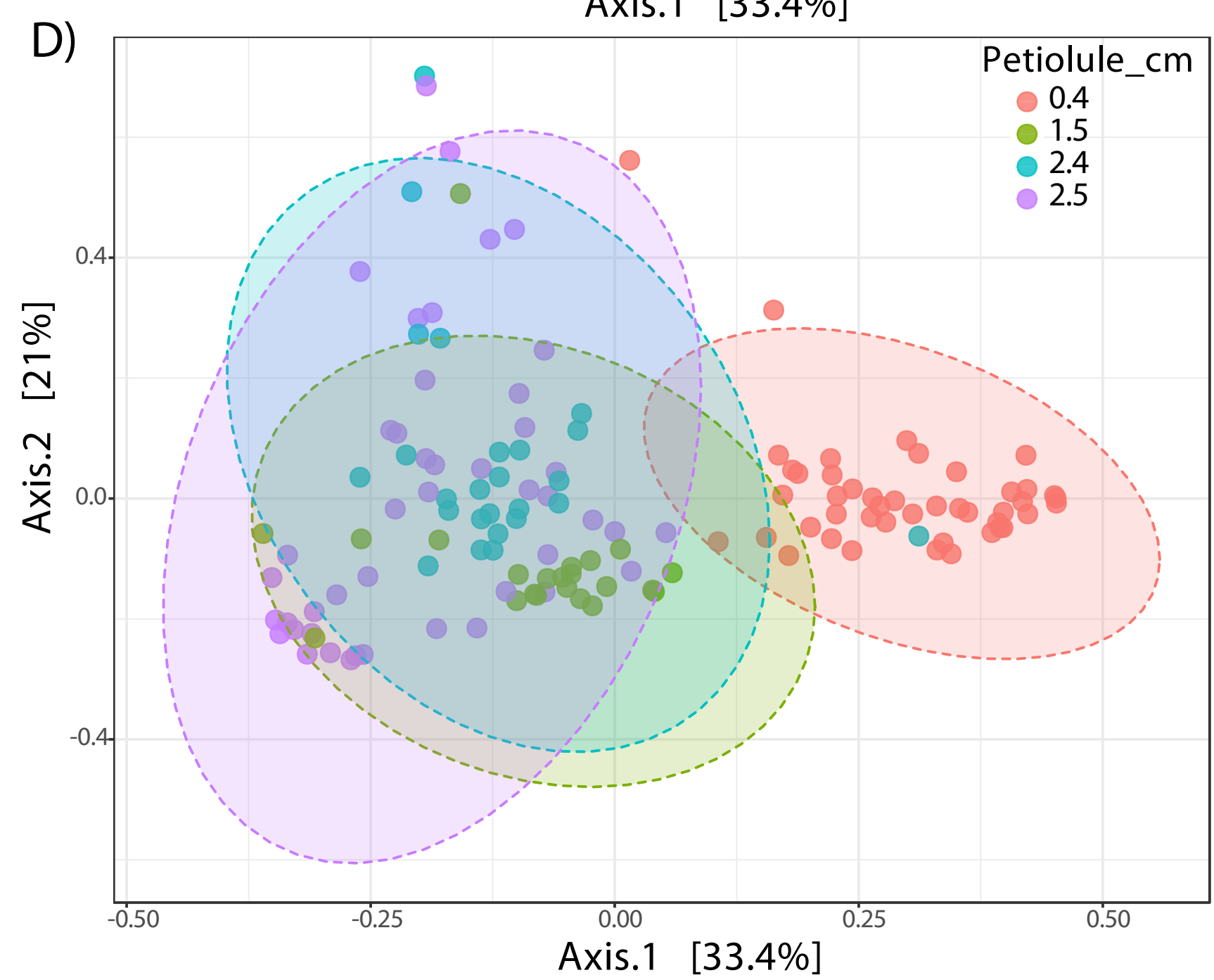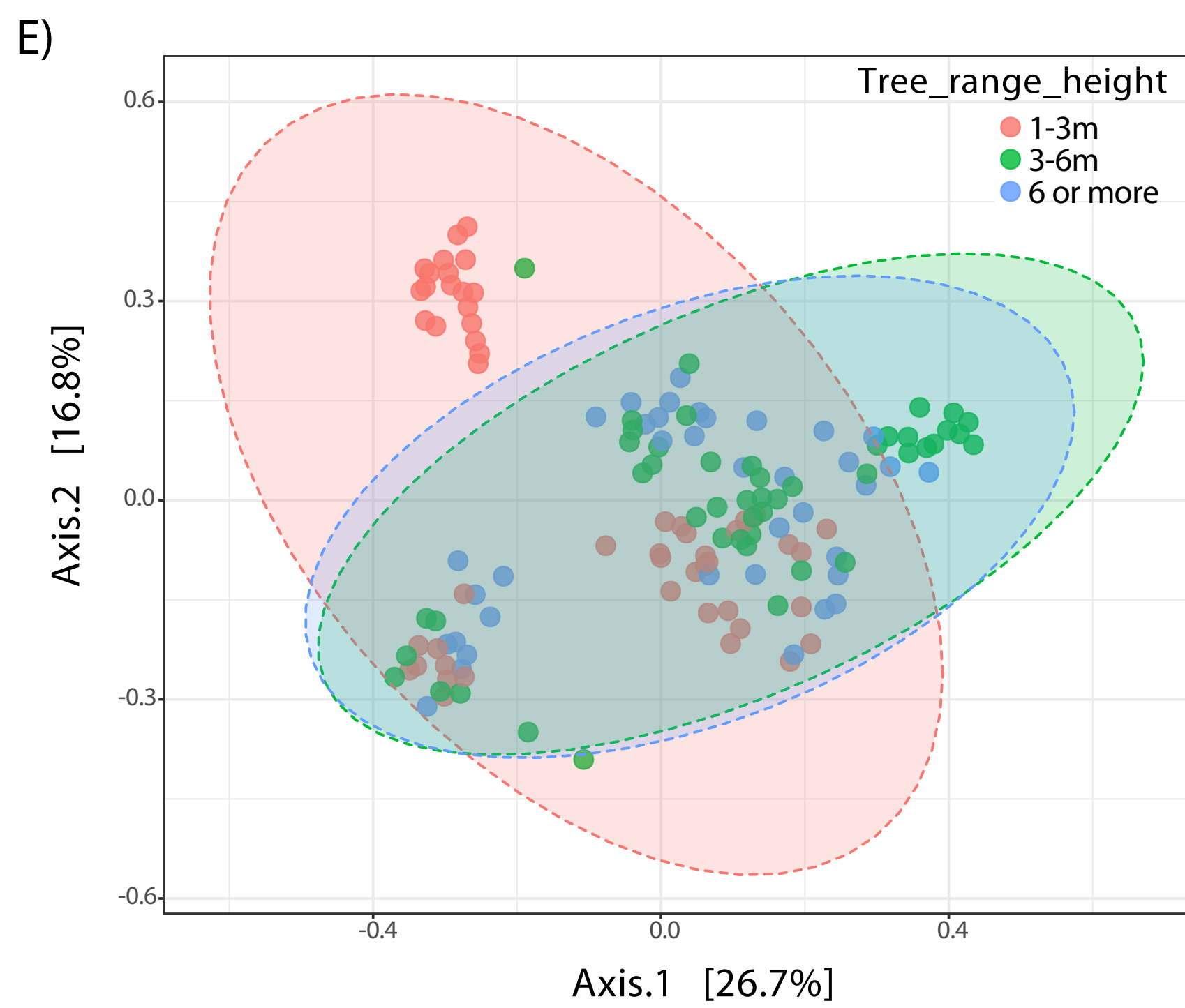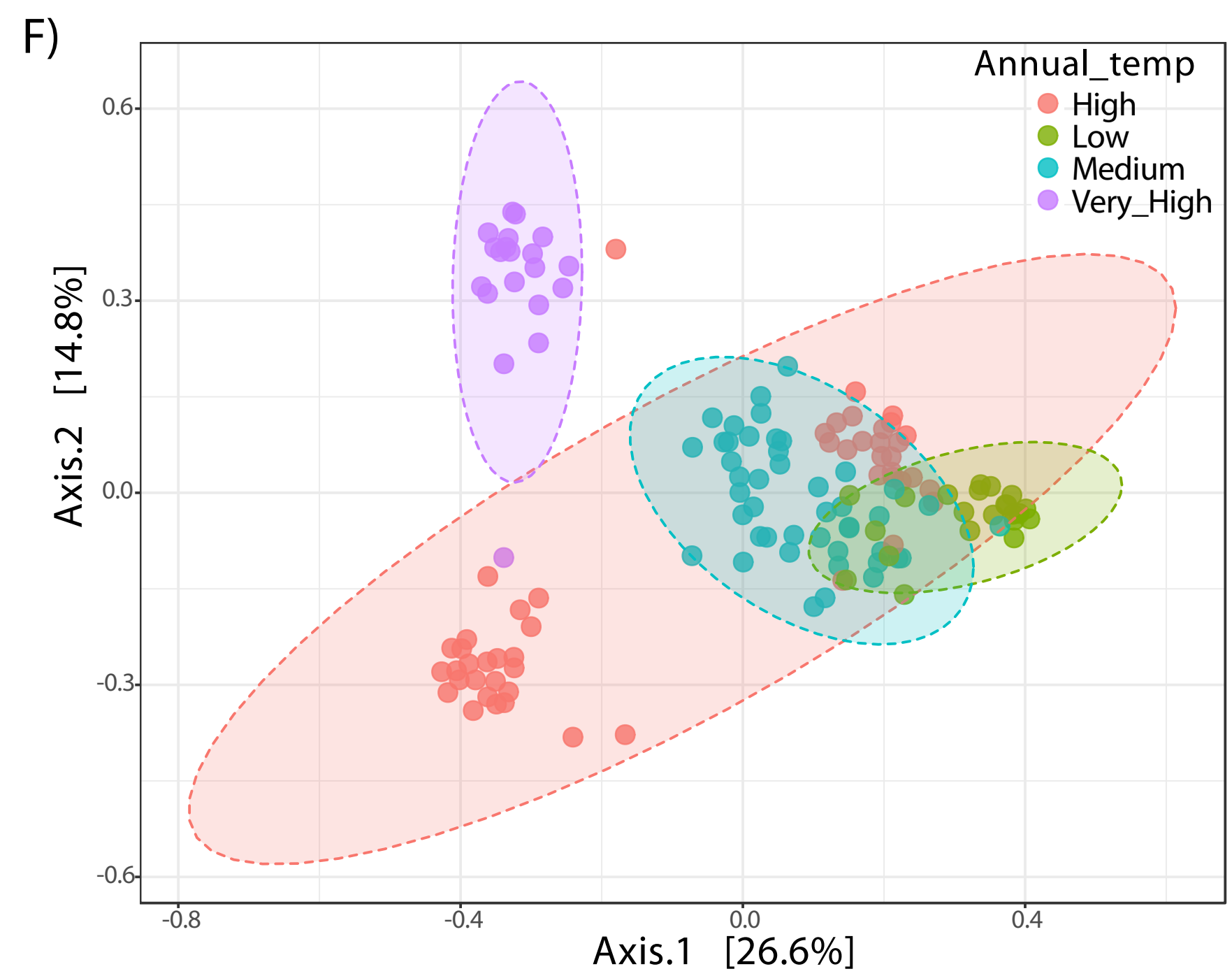

Supplement: S3 Fig — Panel A shows microbiomes according to leaflet number; panel B compared leaflet shape, panel C shows apex morphology, panel D shows petiole length, panel E shows a range of tree height and Panel F displays microbial communities based on mean annual temperature. (PDF) [file pone.0231083.s004.pdf]

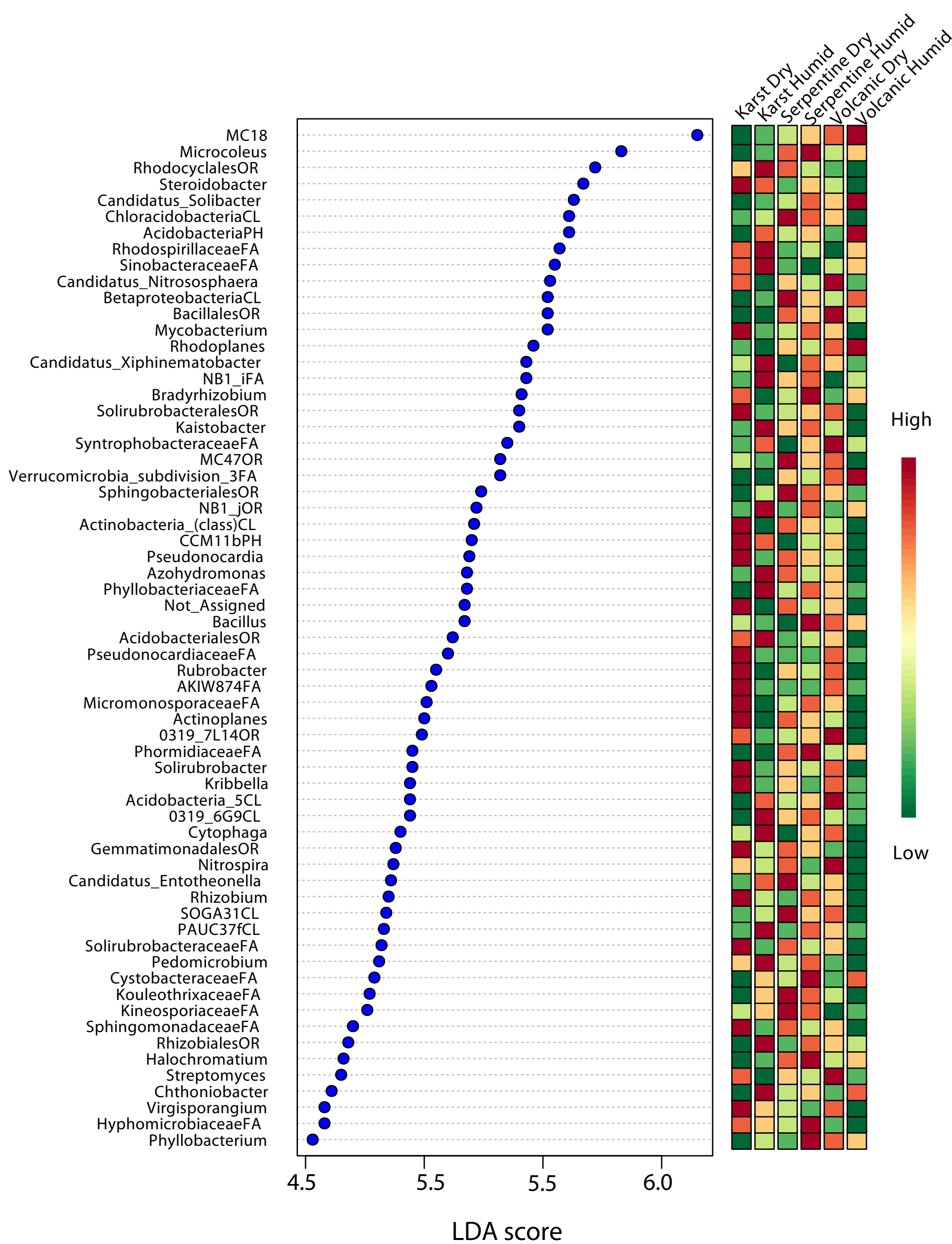

Supplement: S5 Fig — (PDF) [file pone.0231083.s006.pdf]

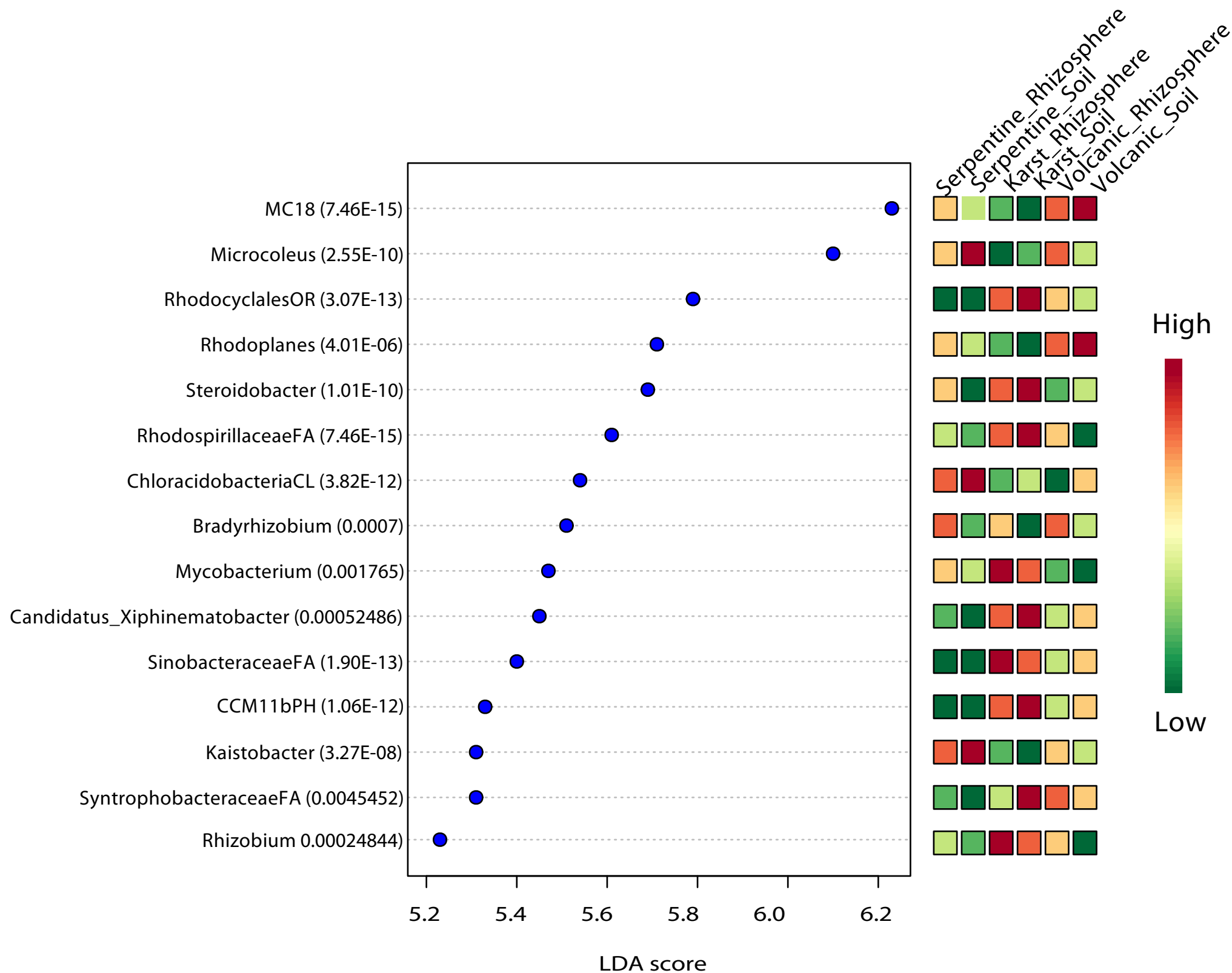

Supplement: S6 Fig — (PDF) [file pone.0231083.s007.pdf]
