# Supplementary material for: Geology and climate influence rhizobiome composition of the phenotypically diverse tropical tree Tabebuia heterophylla
Source: PLoS One. 2020 Apr 7;15(4):e0231083. doi: 10.1371/journal.pone.0231083 (PMC7138329; doi:10.1371/journal.pone.0231083)
Supplement: S4 Fig — The algorithm employs non- parametric Kruskal-Wallis rank sum test to detect phyla with significant differential abundance for a given category (mean annual temperature). (PDF) [file pone.0231083.s005.pdf]

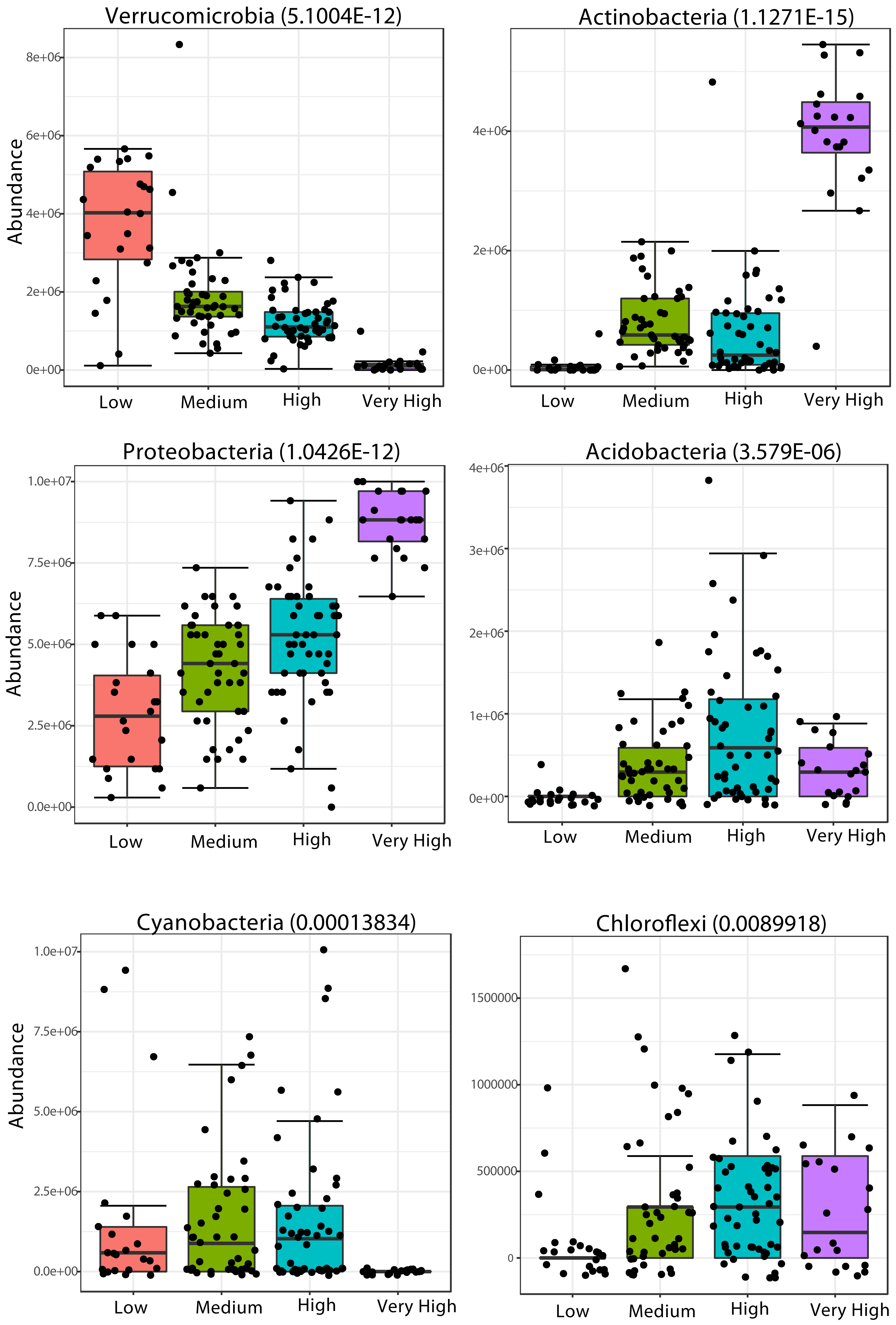

Verrucomicrobia (5.1004E-12)
Actinobacteria (1.1271E-15)
Proteobacteria (1.0426E-12)
Acidobacteria (3.579E-06)
Cyanobacteria (0.00013834)
Chloroflexi (0.0089918)
Abundance
Low
Medium
High
Very High
0e+00
2e+06
4e+06
6e+06
8e+06
0.0e+00
2.5e+06
5.0e+06
7.5e+06
1.0e+07
1e+06
3e+06
0
500000
1000000
1500000
